# Is working from home the new workplace panacea? Lessons from the COVID-19 pandemic for the future world of work

**DOI:** 10.4102/sajip.v47i0.1883

**Published:** 2021-06-21

**Authors:** Jeremias J. de Klerk, Mandi Joubert, Hendrikjan F. Mosca

**Affiliations:** 1 Faculty of Economic and Management Sciences, University of Stellenbosch Business School, Bellville, South Africa

**Keywords:** remote work, work from home, flexible work arrangements, employee engagement, employee experience, COVID-19, lockdown

## Abstract

**Orientation:**

The COVID-19 pandemic has forced millions of employees to work from home as governments implemented lockdowns.

**Research purpose:**

This study examined the impact of working exclusively from home on employee engagement and experience, and determined beneficial and distracting factors.

**Motivation for the study:**

Remote working trends have risen steeply since the onset of COVID-19 and are unlikely to taper off soon. Organisations need to understand the impact of remote work when reconsidering working arrangements.

**Research approach/design and method:**

A dual-approach qualitative design was followed. The sample comprised 25 employees (*N* = 25) who were forced to work exclusively from home during COVID-19. Data were collected through semi-structured interviews.

**Main findings:**

Working from home for protracted periods rendered paradoxical outcomes. Employees could work effectively with improved employee engagement and experience, but there were challenges rendering adverse effects. The experienced benefits of working from home created expectations that this practice would continue in future, along with some office work.

**Practical/managerial implications:**

Organisations need to continue, though not exclusively, with work-from-home arrangements. The ideal ratio of remote work to office work was seen as two to three days per week. However, support and cultural practices would have to be put in place.

**Contribution/value-add:**

The COVID-19 lockdown provided a unique environment to study remote work. For the first time, employees and organisations were placed in a situation where they could experience working from home in a stark and compulsory form, devoid of idealistic fantasies or romanticism.

## Introduction

Since the onset of the coronavirus disease 2019 (COVID-19) pandemic in the year 2020, millions of employees globally have been forced to work from home as governments of many countries implemented various levels of lockdown restrictions. Before the pandemic, organisations adopted work-from-home practices purely as a matter of convenience and to provide conducive working conditions. However, in the year 2020, most governments enforced national lockdowns, which obliged organisations to implement work-from-home arrangements (He, 2020). Early indications suggest that employees would like this ‘new norm’ to continue after the pandemic abates (Iometrics & Global Workplace Analytics [GWA], 2020).

Working from home, or remote work, is a flexible working arrangement (FWA), which consistently correlates with important organisational benefits, such as improved employee engagement and performance (Conradie & De Klerk, 2019; Lee, 2018; Rudolph & Baltes, 2017), and reduced absenteeism (Schaufeli, 2013), enhanced financial returns and organisational effectiveness (Khodakarami & Dirani, 2020). However, extensive remote working can also yield disadvantages, such as social isolation and reduced employee engagement (Sardeshmukh, Sharma, & Golden, 2012; Vander Elst et al., 2017). The concept of employee experience is another useful framework to consider employees’ holistic perceptions of employment relationships (Plaskoff, 2017) and is related to employee engagement practices (Pendell, 2018).

Survey polls relate working from home during the COVID-19 pandemic with increased employee engagement and expectations to continue this praxis (Iometrics & GWA, 2020; Peppercomm Institute for Public Relations, 2020). However, these results have not been tested with scientific rigour. It is thus risky to use them in making decisions for future work arrangements. There is a clear need to determine scientifically the effect of enforced and exclusive remote work on employee engagement and employee experience, as experienced during the COVID-19 pandemic, in order to plan for future work modes.

## Literature review

### The 2020 COVID-19 pandemic lockdowns and remote work

The coronavirus disease 2019 was first identified in China in December 2019, which spread rapidly across the world causing a global pandemic (World Health Organization [WHO], 2020). By mid-January 2021, the number of COVID-19 cases had already reached 95 million globally, resulting in two million deaths, with cases continuing to rise exponentially (WHO, 2020). In order to curb the spread of COVID-19, most countries, including South Africa, introduced lockdowns. Depending on the country, lockdown included the closure of many facilities and restriction on leaving home for non-essential work. Organisations had to find ways to keep their businesses afloat and employees productive, resulting in a marked shift to work-from-home practices. According to the global survey of Iometrics and GWA (2020), 88% of respondents were working from home regularly in 2020. By early 2021, employees in most countries, including South Africa, were still required or encouraged to work from home, where possible.

The general media frequently speculate about the outcomes and future of working from home. One of the polls revealed that 74% of chief financial officers intended to shift some employees permanently to remote work, mostly to save costs (Lavelle, 2020). Another poll revealed that 77% of respondents were satisfied with flexibility in balancing work and non-work activities, and that 69% were satisfied with overall productivity, well-being, engagement and a feeling of safety, depending on the home environment and resource availability (Iometrics & GWA, 2020). The poll further indicated that 76% of respondents wished to work from home at least 1 day per week after the lifting of lockdown, with 16% not wanting to return to office at all. It is reasonable to expect that these work-from-home experiences will have a far-reaching effect on how remote work is viewed and managed in future.

### Flexible working arrangements, remote work and working from home

Flexible working arrangements provide flexibility in terms of the place of work (working from home), time of work (flexible hours) and continuity of work (breaks in work activity) (Ten Brummelhuis, Bakker, Hetland, & Keulemans, 2012). As work-from-home arrangements do not necessarily prescribe when employees should work, working hours will inevitably be affected as employees have the flexibility to work at times that suit their personal schedules.

Flexible working arrangements have been associated with improved employee health and well-being, improved management of work and family role conflicts (Mache, Servaty, & Harth, 2020; Rudolph & Baltes, 2017), increased engagement (Anitha, 2014; Conradie & De Klerk, 2019; Rudolph & Baltes, 2017; Ten Brummelhuis et al., 2012), greater job autonomy and psychological resources (Mache et al., 2020) and improved commitment. Similarly, remote work has been correlated with outcomes, such as higher productivity, as employees are willing to recommit part of the time saved by not having to commute (McNaughton, Rackensperger, Dorn, & Wilson, 2014), improved morale (Boell, Campbell, Cecez-Kecmanovic, & Cheng, 2013), fewer work interruptions (Gajendran & Harrison, 2007; Kazekami, 2020; McNaughton et al., 2014), better ability to coordinate work and non-work commitments (Boell, Cecez-Kecmanovic, & Campbell, 2016), greater job satisfaction and commitment (Gajendran & Harrison, 2007; Kazekami, 2020), less role stress and work–family conflict (Masuda, Holtschalg, & Nicklin, 2017) and increased work–life balance (Boell et al., 2013).

In contrast, the challenges of extensive remote working include reduced teamwork and collaboration (Boell et al., 2013), work–life blurring (Eddleston & Mulki, 2017), increased isolation and lack of meaningful connections with others (McNaughton et al., 2014; Vander Elst et al., 2017), overwork, infringement on family roles and work–family conflict (Eddleston & Mulki, 2017), distractions, loneliness, worry and guilt (Mann & Holdsworth, 2003), increased stress and decreased life satisfaction (Kazekami, 2020).

Company culture has a significant effect on the outcomes of flexible working arrangements (Putnam, Myers, & Gailliard, 2014). A culture that is not supporting and trusting can result in unrealistic organisational expectations and demands on remote workers, which can lead to tension and unhealthy work practices (Perlow & Kelly, 2014). Despite the benefits of remote working, concerns about remote workers’ lack of physical presence and connection to the organisation continue to surface (Belle, Burley, & Long, 2015). Working from home is more than a change in physical location; it also alters the physical environment of the worker, including the equipment, tools and resources required to complete tasks and the nature of interactions with other employees (Sardeshmukh et al., 2012). Working from home changes the nature of work and human engagement in work processes (Boell et al., 2013). Moreover, it can be argued that mandatory working from home during lockdown is a flexible spatial option in form, but not in spirit, because it is missing the element of choice and may not deliver the same benefits as voluntary working from home (Hyatt & Coslor, 2018).

### Employee engagement, outcomes and antecedents

The challenges of COVID-19 for businesses have amplified the importance of having engaged employees survive and prosper during these difficult times. Employee engagement can be defined as ‘a positive, fulfilling motivational state of work-related well-being’ (Wood, Oh, Park, & Kim, 2020, p. 242). Employee engagement has consistently been correlated with several important organisational outcomes (Bailey, Madden, Alfes, & Fletcher, 2017) that constitute a competitive advantage (Schneider, Yost, Kropp, Kind, & Lam, 2018), such as enhanced commitment and performance, reduced absenteeism (Schaufeli, 2013), enhanced organisational financial returns and organisational effectiveness (Khodakarami & Dirani, 2020) and positive financial and customer metrics (Bailey et al., 2017; Schneider et al., 2018).

In their definition of employee engagement, Schaufeli, Salanova, Gonzalez-Roma and Bakker (2002) distinguished three dimensions: *vigour* (high levels of energy and mental resilience, a willingness to invest effort in work), *dedication* (a strong psychological involvement in one’s work, a sense of meaning, enthusiasm, inspiration, pride and challenge) and *absorption* (immersion in one’s work, being completely focused and happily engrossed in work). Kahn (1990) posited that employee engagement is the outcome of psychological safety, available job resources and psychological meaningfulness of the work. These antecedents have been linked consistently with employee engagement (Anitha, 2014). Positive antecedents, such as job resources, positive psychological states and positive leadership and perceived organisational support are routinely linked with increased levels of engagement (Bailey et al., 2017; Khodakarami & Dirani, 2020), whereas aspects such as abusive supervision are linked with decreased levels of engagement (Bailey et al., 2017).

The job demand-resources (JD-R) framework explains employee engagement as the outcome of a balance between job demands and job resources (Bailey et al., 2017; Lee, 2018). Job demands refer to ‘[t]hose physical, psychological, social or organisational aspects of the job that require sustained physical and/or psychological (i.e. cognitive or emotional) effort’ (Schaufeli & Bakker, 2004, p. 296). Job resources are:

[*T*]hose physical, psychological, social or organisational aspects of the job that either/or (1) reduce job demands and the associated physiological and psychological costs; (2) are functional in achieving work goals; (3) stimulate personal growth, learning and development. (Schaufeli & Bakker, 2004, p. 296)

High job resource availability fosters higher levels of employee engagement (Rudolph & Baltes, 2017; Schaufeli & Bakker, 2004), whereas, a lack of resources prevents employees or excessive job demands and fosters mental withdrawal or disengagement (Schaufeli & Bakker, 2004).

The JD-R model is primarily used to investigate both the positive and negative relationships between work engagement and family-related factors (Wood et al., 2020). By applying the JD-R model, Bakker, Ten Brummelhuis, Prins and Van der Heijden (2011) demonstrated that the combination of substantial job demands, such as workload, and deficient job resources, such as supervisory support, correlates with work-home interference (WHI). Work-home interference is a common issue for employees, because most people have considerable family responsibilities in addition to their work demands. However, although work overload is an important antecedent of WHI, it does not necessarily result in WHI when employees experience sufficient resources (Bakker et al., 2011). Research has shown that job resources, such as supportive supervisors, play significant roles in enhancing work engagement and reduce WHI (Wood et al., 2020). In contrast, a scarcity of resources combined with demands, such as the conflict between work and family roles, promote WHI and tend to erode work engagement and well-being (Wood et al., 2020). An individual’s work environment can have a substantial influence on his or her non-work-related life situations, and the other way around, with both positive and negative effects on the individual’s well-being (Wood et al., 2020). Flexible working arrangements, such as remote work, signal a supportive working environment in which employees are cared for by their organisation (Wood et al., 2020). Remote work offers employees a level of control over their place of work (and, in practice, also time), providing a sense of psychological job resources.

Conservation of resources (COR) theory (Hobfoll, 1989, 2002) describes how people react to the stressors in their environment that might influence their well-being. People draw on resources to cope with the stressors. If one still cannot cope, or if too many resources are consumed in the process, it will result in stress (Hobfoll, 2002). Hobfoll (2002) made the distinction between contextual resources and personal resources. Personal resources are inherent to the individual and include aspects such as personal characteristics, time availability and physical energy (Hobfoll, 2002). Contextual resources are located outside the self and can be found in the individual’s social context, such as one’s working environment at home, or the support provided by one’s manager. Ten Brummelhuis and Bakker (2012) applied COR (Hobfoll, 1989, 2002) to develop the work–home resources (W-HR) model to gain insights into how personal resources interact with demanding aspects of the work domain. Working-from-home relates to both contextual and personal resources. For instance, increased autonomy (a contextual resource resulting from working from home) can be used to reschedule work in order to accommodate an individual’s family-time requirements (a personal resource) (Ten Brummelhuis & Bakker, 2012). Both work and home domains potentially could augment personal and contextual resources, which can be used to improve home and work outcomes (Ten Brummelhuis & Bakker, 2012). In contrast, the W-HR model also describes how the conflict between work and home demands can deplete personal resources. For instance, family time demands, such as home schooling as a result of working from home, may require the individual to continue working late at night to cope with work demands (Du, Bakker, & Derks, 2020). This situation depletes an individual’s personal resources, eventually contributing to role overload and burnout, which is likely to put a strain on contextual resources (Aw, Ilies, Li, Bakker, & Liu, 2021; Du et al., 2020).

Psychological detachment (an individual’s sense of being away from the work environment) represents an important psychological mechanism that enables employees to recover from their work-related effort (Bakker, Du, & Derks, 2019). Switching off from work-related issues through psychological detachment enables people to stop consuming the personal resources required to be effective at work. Having personal resources available, then prevent negative spill-over from work to home and promote the restoration of depleted personal resources (e.g. engagement) to positively influence well-being (Bakker et al., 2019). It could thus be reasoned that being away from the work environment when working from home would promote psychological detachment and augment personal resources. However, Bakker et al. (2019) found that regular rumination about work-related matters when working from home erodes the ability for psychological detachment and effective use of personal resources. The long-term effect of working from home on personal resources and well-being is thus not clear and requires further investigation.

### Employee experience

The concept of employee experience originated from the thinking used in customer experience (Plaskoff, 2017) and is increasingly viewed as a critical human capital trend (Walsh & Volini, 2017). Although little scientific research has been conducted on employees’ experience, improved employees’ experience has been linked to benefits, such as improved productivity and revenue, improved employee engagement and customer satisfaction (Hektner, Schmidt, & Csikszentmihalyi, 2019) and attracts a better talent pool (Van Vulpen, 2019).

Employee experience can be defined as the employees’ set of perceptions about their work experiences in response to interactions with the organisation (Plaskoff, 2017) and relates to the relationship between work complexity and behavioural norms, such as collaboration and empowerment (Dery & Sebastian, 2017). Morgan (2015) suggested a three-domain framework for employee experience: cultural, physical and technological environments. The cultural environment is about how employees experience the people in an organisation and how things are performed (Morgan, 2015). The physical environment constitutes the tangible elements at the workplace with which employees interact. The technological environment entails all the tools available to carry out the work.

Improving employees’ experience starts by understanding their needs and developing a culture, in which employees can reach their full potential (Plaskoff, 2017) and find meaning (Lemon, 2019). When organisations understand the employees’ needs, it helps them to understand what is of importance at particular points in employees’ journeys (Nelson & Neicu, 2020; Plaskoff, 2017).

### COVID-19, working from home, employee engagement and employee experience

Although working from home has become the standard of working for millions, there is little previous research on how working from home influences employee engagement and employee experience (Masuda et al., 2017). Indeed, Vander Elst et al. (2017) suggested that the disadvantages inherent in extensive working from home may exceed the associated advantages. Whilst FWAs have previously been linked with employee engagement, it is uncertain how working from home during COVID-19 has affected employee engagement and how this experience might influence the future of remote working. For instance, just as working far away from one’s home is likely to result in homesickness and ensuing depletion of personal resources (Du, Derks, & Bakker, 2018a), one might expect that ruminative thoughts about the work environment (Du et al., 2018b) when separated from the workplace for a prolonged period of time when working exclusively from home may result in a similar experience. However, it is largely unclear how enduring separation from the workplace might influence employees’ work experience. This study aims to explore the impact and experience of forced flexible work arrangements on employee engagement and employee experience during the COVID-19 pandemic and to appraise employee expectations of flexible work arrangements in a post-pandemic future.

## Research design and method

The research study combined a double qualitative study conducted by two independent researchers using semi-structured interviews to control for self-selection bias, minimising sampling bias, researcher bias and interview bias as discussed under the section on strategies to ensure trustworthiness.

### Research setting and sampling

The research study was conducted during the initial South African COVID-19 lockdown period from 27 March 2020 to 17 August 2020, when working from home was strictly enforced. A dual-frame sampling approach was used. Firstly, convenience sampling was used, comprising members of a social media group (Facebook) with 3700 members. An invitation to participate in the research was posted on the platform. The criteria to take part in the study were that participants needed to have worked from home exclusively, at least during level 5 of the lockdown, but not before the lockdown. In addition, a purposive sampling approach was conducted to minimise self-selection bias by identifying a shortlist of potential participants from the researchers’ LinkedIn profiles, who showed a good fit with the criteria. Eventually, 25 participants were interviewed, of which 14 from the Facebook sampling process and 11 participants from the LinkedIn process.

Most participants (52%, *N* = 13) were between the ages of 30 and 40 years, 24% (*N* = 6) of participants were between 20 and 30 years, 20% (*N* = 5) were between 40 and 50 years and 4% (*N* = 1) were between 60 and 70 years. The sample group consisted of six men (24%) and 19 women (76%). Most participants described their position in the organisation as middle management (44%, *N* = 11), followed by junior management or professional (32%, *N* = 8), senior management (20%, *N* = 5) and executive management (4%, *N* = 1). Participants represented 14 different industries, with the largest representation from financial services (24%, *N* = 6), transportation (12%, *N* = 3) and construction (8%, *N* = 2). Participants represented a wide variety of professions, such as accounting, information and telecommunication, production, environmental science, financial management, public relations, marketing, legal, project management, property valuation, human resources and tertiary education.

### Data collection and recording

Semi-structured interviews were carried out for data collection, and interview guides were developed to ensure trustworthiness of the data collection process. The first set of questions were related to the interviewee’s remote working experiences during working from home. The second set of questions were related to employee engagement and employee experience as discussed in the literature review. Interviews were conducted through Microsoft Teams or Zoom because of lockdown restrictions. After each interview, recordings were transcribed using either the Microsoft Word transcribe function or Otter.ti software, followed by manual checking and corrections where necessary.

### Strategies employed to ensure trustworthiness

Combining purposive sampling with convenience sampling contributed to controlling for self-selection bias and minimising sampling bias. Two researchers developed their interview guides independently to minimise researcher and interview bias. The research study was conducted whilst participants were still subject to working from home, preventing wishful ideations of what working from home could be like, or post-hoc recollections.

Care was taken not to draw any conclusion whilst doing the interviews or transcriptions, but to let themes emerge from a structured thematic analysis using Atlas.ti software. ATLAS.ti has the benefit of speed, consistency, rigour and access to analytical methods not available by hand (Given, 2008). The findings were triangulated, as the two sets of data gathered by the respective researchers were independently transcribed and analysed by them before combining common sub-themes and themes from their analyses.

### Data analysis

A thematic analysis approach (Braun & Clarke, 2006) was followed to analyse the data rigourously and systematically. This technique began with correcting the electronic transcriptions, which allowed for building familiarity with the data (Saunders, Saunders, Lewis, & Thornhill, 2011), followed by organising the textual data thematically through a process of coding, axial coding and thematic analysis (Cassell, 2015) to arrive at the key sub-themes and themes. The software package, ATLAS.ti, was used for analysing the qualitative data.

### Ethical considerations

This research study was approved by the Research and Ethics Committee (Social, Behavioural and Education Research) of Stellenbosch University.

## Findings

The key sub-themes and themes of the thematic analysis were identified from the interviews (Table 1), which are discussed later. Although these themes and sub-themes represent a useful heuristic categorisation of the findings, they are interdependent and not neatly separable. Overlap in the discussions is thus unavoidable.

**TABLE 1:** Key themes and sub-themes of the thematic analysis.

Key themes	Sub-themes
People can effectively work from home, but not without challenges.	1.1. Work-from-home realities are not necessarily aligned with expectations. 1.2. Job demands, personal and contextual resources and demands significantly influence work-from-home experiences.
Working from home renders paradoxical outcomes.	2.1. Increased flexibility renders appreciated benefits, but blurs boundaries and makes work-life balance difficult. 2.2. Working from home provides some organisational benefits, but reduces collaboration.
Working from home influences employee engagement and employee experience, but not uniformly.	3.1. Energy, enthusiasm and immersion increase for some, but decrease for others. 3.2. Working from home influences employee experience in all three of its domains: cultural, physical and technological. 3.3. Being forced to work from home influences engagement and employee experience.
Work-from-home experiences created expectations of continuing with this praxis in future.	4.1. The perceived effectiveness of virtual working created a new norm. 4.2. Employees want and expect to continue with work-from-home arrangements, but not exclusively so.

### Theme 1: People can work effectively from home, but not without challenges

#### Sub-theme 1.1: Realities of working from home are not necessarily aligned with expectations

Work-from-home arrangements were initially received with enthusiasm and excitement, with idealisations about the convenience of not having to go to work and the flexibility to do other things besides work. However, after the initial excitement subsided and reality kicked in, the realised experience was not always positive. Participants, especially those who had not previously worked from home, underestimated the adverse experiences that inevitably accompany extensive remote working:

‘At the beginning it was quite fun to be at home and it really has gotten more and more difficult.’ (P1, 30–40, middle management)

‘Working from home has been probably one of the most challenging things that I have gone through in a very, very long time.’ (P14, 30–40, professional)

The work-from-home experience was not merely a continuation of a normal working environment from a remote location, but was accompanied by significant experiences of discomfort and uncertainty:

‘For people who are used to fieldwork and meeting other people and being out and about, the cabin fever was probably worse than for most other professions.’ (P9, 60–70, middle management)

These findings confirm that working from home can be motivating, but an exclusive work-from-home arrangement over a long period can be demotivating. The following discussions provide insights into the reasons underlying this finding.

#### Sub-theme 1.2: Job demands, personal and contextual resources and demands significantly influence experiences

Participants with previous work-from-home experiences were more likely to have a positive work-from-home experience. Contextual resources (Ten Brummelhuis & Bakker, 2012), such as an appropriate room or private space for use as an office, are essential. More than 70% of participants either had a separate room to use as an office or created one. In contrast, eight participants mentioned that they had to set up an office in a communal space, such as the dining room, which was not ideal:

‘You cannot now just work from the dining room table; it doesn’t work. You have to have that separate … It also helps with making sure that you keep that separation.’ (P1, 30–40, middle management)

As working from home continued, participants invested in setting up dedicated office spaces and resources to ensure an effective work-from-home space. The findings confirm the importance of the job demands and resources model; sufficient contextual job resources are essential to cope with personal and work demands (Rudolph & Baltes, 2017; Ten Brummelhuis & Bakker, 2012).

Several participants attributed effective work-from-home practice to experiencing fewer interruptions at home. Whereas constant interruptions at the office distract, work-from-home arrangements provided quiet spaces where participants could focus and be productive. However, some participants experienced significant interruptions whilst working from home, for example, from garden services or constant barking of neighbours’ dogs, which adversely affected their work-from-home experience:

‘There are things that can distract you, that can make you less productive than what you think you should be.’ (P3, 20–30, middle management)

These findings confirm that contextual demands in the face of low contextual resources can deplete personal resources (Ten Brummelhuis & Bakker, 2012). It also confirms previous research findings (Gajendran & Harrison, 2007) that although some individuals enjoy fewer interruptions at home and may have increased job satisfaction, others may be subject to more distractions, depleting personal resources and decreasing job satisfaction.

Lockdown restrictions initially required the closure of schools and crèches, disabling parents’ access to their accustomed support structures. Participants with children, especially women, experienced significant distractions, accompanied by increased stress and anxiety and long working hours during this time:

‘If obviously you have to spend a few hours with your kids … then you have to try and make those hours up at night.’(P10, 30–40, professional)

However, when lockdown regulations relaxed, schools reopened and childcare in the form of schooling became readily available again. Their children’s school attendance provided participants with relief from parenting duties and a significant improvement in their work-from-home experience:

‘I’m lucky now because my kids are back at crèche. So, yeah, that I think is my biggest thing is that there’s not those other distractions and also you can be really effective.’ (P10, 30–40, professional)

These findings support those of previous research, which suggest that remote working may infringe on family roles and increase work–family conflict (Eddleston & Mulki, 2017). Again, these findings confirm that contextual demands in the face of low contextual resources tend to deplete personal resources (Ten Brummelhuis & Bakker, 2012). As school closure was distinct to the COVID-19 lockdown, this distraction will not specifically be considered further in themes and future recommendations for the purposes of this study. However, it remains an issue that requires much attention and research.

Work-from-home experiences largely depended on how participants perceived being supported by their organisations and managers. Organisational support signalled messages of care and assurance that well-being of staff was important:

‘A lot of this is dependent on the company you work for, and how they treat you, and how they react to everything, and I can just be quite grateful for being part of a company that reacted well, listens to the complaints, [*and*] addresses them.’ (P1, 30–40, middle management)

‘They’ve delivered office chairs to our homes. If you needed headsets, they would deliver it to your door. … that mind-set that came from them helped to know that you have their full support.’ (P2, 30–40, middle management)

Organisational culture played a significant role in employees’ experience as a contextual resource (Ten Brummelhuis & Bakker, 2012), and working from home was positively experienced in a trust-based culture:

‘If your manager is a very trusting and open person and they lead by … then they’re very open. So, that helps you to be a bit more at ease in terms of the flexible type of working from home.’ (P12, 40–50, senior management)

Lack of trust and an unsupporting work culture, however, extracted longer hours of employees, which, in turn, led to stress, anxiety and exhaustion resulting in the depletion of personal resources (Ten Brummelhuis & Bakker, 2012). Some participants reported implicit organisational expectations that they always be available online, sometimes even after hours. Several participants noted perceived pressures of having to respond immediately to mails in order to prove that they were working, and felt that they could not take any break and had to work excessive hours:

‘I feel like I have more anxiety [*now*] that I’m at home … I feel that I can’t go for a walk, that I must be at my computer all the time.’ (P1, 30–40, middle management)

‘There is almost a tendency to work longer hours. You feel the pressure to show that you are delivering.’ (P4, 40–50, professional)

The pressure and expectation to be constantly available, however, diminished as organisations settled into the work-from-home rhythm, with more acceptance that everyone was working, even when not immediately available. This, in turn, significantly boosted employees’ experience:

‘[*N*]ow I love it. I love it. I love it. I love it. It’s just, I feel like we are treated as adults.’ (P19, 30–40, middle management)

Organisational and managerial support had a significant impact on the work-from-home experience and engagement of employees. This finding confirms that of previous research, suggesting that organisational support results in increased levels of perceived job resources (Khodakarami & Dirani, 2020; Ten Brummelhuis & Bakker, 2012) and work engagement (Bailey et al., 2017; Wood et al., 2020).

#### Concluding reflections – Theme 1

The findings under theme 1 confirm that although working from home can be motivating and promote engagement (Conradie & De Klerk, 2019), an exclusive work-from-home arrangement that extends over a long period tends to generate negative outcomes and can be demotivating. The latter finding is a new contribution that was enabled by the COVID-19 lockdown situation, as previous studies could not investigate the impact of working from home for an extensive period of time. The findings suggest a work-related version of ‘home-sickness’ (Du et al., 2018b) when working from home extensively and exclusively, which is a new contribution. The findings also confirm the importance of organisational and managerial support (contextual resources) (Ten Brummelhuis & Bakker, 2012) on the work-from-home experience to increase levels of personal resources (Ten Brummelhuis & Bakker, 2012), which, in turn, promote engagement and well-being (Wood et al., 2020). Augmenting contextual and personal resources is important to obtain the maximum benefit from the working-from-home experience.

### Theme 2: Working from home renders paradoxical outcomes

#### Sub-theme 2.1: Increased flexibility renders appreciated benefits, but blurs boundaries and makes work–life balance difficult

Working from home enabled participants to better manage their personal lives, allowing them the flexibility to attend to personal commitments during the day:

‘I can take my son to his extramural activity and I can sit in the car and work. … I literally just take my laptop and I go and carry on working. But I can watch him. So, it’s that flexibility … just being able to do things like that, which is very cool.’ (P2, 30–40, middle management)

Most participants observed benefits of increased flexibility, experiencing less work pressure and being able to attend to their personal things, contributing positively to improved well-being:

‘I think what I will miss if I had to go back to the office is the flexibility in terms of just taking time out to sort of energise myself … regular breaks, 15 minutes, half an hour; going for a walk. Having a quick power nap to recharge.’ (P8, 40–50, middle management)

Time and cost savings, such as not having to commute to work and not requiring formal work attire, were seen as major work-from-home benefits. Working from home also resulted in participants being more relaxed, and experiencing reduced strain on personal relationships and improved work–life balance and well-being:

‘You inevitably save money by working from home because you’re not travelling, so it’s not the petrol, you don’t tend to then buy lunch … or clothes. I’m now in a tracksuit and leggings all day, all week.’ (P1, 30–40, middle management)

‘[*Previously*] I only saw my daughter 15 minutes in the evening when I got home … then my husband has to do everything, and that also puts strain on a marriage [*and*] on your relationship.’ (P5, 30–40, middle management)

These findings confirm those of previous research (Rudolph & Baltes, 2017), which found that employees who work remotely are likely to be satisfied and less likely to experience work–family conflict. These benefits promote the experience of increased personal and contextual resources (Ten Brummelhuis & Bakker, 2012). Paradoxically, the flexibility and pressures that inherently accompany working from home blurred work and home boundaries and made prioritising and work–life balance difficult:

‘The line between work and personal life became very blurry.’ (P22, 30–40, junior management)

The absence of natural daily structures made it complicated to distinguish between work and personal life and to create distance between home and work, making it challenging to maintain a healthy work–life balance:

‘The downfall to that is then 10, 11 o’clock at night, you’re still sitting behind your laptop … the balance is a little bit difficult.’ (P14, 30–40, professional)

‘It’s difficult, because you work at the place where you sleep.’ (P24, 20–30, junior management)

A noteworthy finding was that although commuting was generally frustrating, it played an important role in creating a tangible boundary between work and home:

‘The drive home from work actually forced your brain to switch off. Sometimes we left our laptops at work when the work wasn’t that tough. So it was a deliberate break that you’ve had to take.’ (P21, 20–30, senior management)

This finding confirms that working from home makes psychological detachment from work difficult (Du et al., 2018b) and this tends to deplete personal resources.

These findings confirm that working from home presents complex paradoxes to setting priorities and work–life balance, confirming the previous research findings (Kazekami, 2020; Vander Elst et al., 2017) that although remote working may have assisted employees in combining their work with their family roles, blurred boundaries between work and family life made psychological detachment from work difficult.

#### Sub-theme 2.2: Working from home provides some organisational benefits, but reduces collaboration

Notwithstanding the difficulties discussed above, most participants experienced an increase in productivity and efficiency. Working online facilitated opportunities and benefits, which were previously unthinkable:

‘The company themselves … have told us they’ve realised how productive people can be at home.’ (P1, 30–40, middle management)

‘I get more done in a half-day than I used to get done at the office.’ (P13, 30–40, middle management)

Although several participants observed distractions and disruptions at home, paradoxically they also reported fewer disruptions than at the office and felt that they had been more productive:

‘I find I work much more efficiently, because in the office, we are in an open-plan office and there are these ongoing conversations. Someone’s always on the phone. Someone’s having a chat. Someone is asking a question … I found [*working from home*] to be more efficient.’ (P4, 40–50, professional)

This finding is aligned with previous research findings (Boell et al., 2016; Kazekami, 2020; McNaughton et al., 2014), which suggest that remote work often contributes to higher productivity. Paradoxically, participants missed the efficiency of instant responses obtained by walking to a colleague for a quick discussion. Working from home meant sending a message and waiting until a colleague had time to respond or formally making a phone call or arranging for a discussion. Participants found the absence of instant informal communication frustrating:

‘[*Y*]ou need to make a point of talking to somebody or emailing them or sending them an instant team message, whereas in the past, you could just pop up and say hi, listen … But now it’s actually an effort to talk.’ (P19, 30–40, middle management)

‘Everything just took so long. So, something which would normally take me five minutes to work out took me 10 days to sort out because you have people who don’t respond to emails or WhatsApps.’ (P10, 30–40, professional)

Paradoxically to the improved organisational benefits of working from home, these are not necessarily sustainable over long periods as working from home inevitably fosters the loss of personal interaction and collaboration, a key obstacle bemoaned by all participants:

‘Working from the office, that fosters collaboration … and connection, which is important because we are human beings. We are … wired for connection.’ (P8, 40–50, middle management)

On an emotional level, all participants felt isolated, craving for physical and personal interaction, resulting in negative experiences:

‘I think [*exclusively working from home*] is emotionally and mentally taxing as opposed to anything else. Limited human interaction is very mentally and emotionally demoralising.’ (P14, 30–40, professional)

Insufficient bandwidth prevented the use of webcams, resulting in the loss of non-verbal communication and social cues. This affected social interaction negatively:

‘We don’t put our cameras on when we have meetings, so you don’t pick up on social cues. I’m such a people person and I would like to pick up on your body language.’ (P1, 30–40, middle management)

Work interactions became task focussed, dominated by operational matters, at the cost of connecting personally. Participants increasingly felt lonely and secluded as communication became less relational and more transactional:

‘There isn’t a sense of connection because it’s always focused on operational issues, on work matters, and not … well-being. Where people are at. What are they struggling with? It’s always delivery outputs, KPIs [*key performance indicators*], objectives.’ (P8, 40–50, middle management)

These findings confirm those of previous research (Kazekami, 2020; Mann & Holdsworth, 2003; Vander Elst et al., 2017), which suggested that experiences of isolation and loneliness stemming from a lack of physical interaction are negative emotional outcomes of remote work. It also confirms how the difficulty of personal interaction and collaboration when working from home diminishes contextual resources to deplete personal resources (Ten Brummelhuis & Bakker, 2012).

#### Concluding reflections – Theme 2

The findings under this theme confirm that working from home tend to blur the boundaries between a person’s work life and personal life (Wood et al., 2020), which result in several complex and paradoxical outcomes (Kazekami, 2020; Vander Elst et al., 2017). Although remote working assists employees in combining their work roles with their family roles, blurred boundaries between work and personal lives make setting and keeping priorities and boundaries between work and personal lives very difficult. Moreover, consistent ruminating thoughts about work matters make psychological detachment from work difficult (Du et al., 2018b), which tend to deplete personal resources.

The findings confirm that remote working often contributes to higher productivity (Boell et al., 2016; Kazekami, 2020; McNaughton et al., 2014). However, employees miss and long for the efficiency and intimacy of regular physical interaction and collaboration with colleagues. This finding also confirms that working from home tends to lead to experiences of isolation and loneliness stemming from a lack of physical interaction (Kazekami, 2020; Mann & Holdsworth, 2003; Vander Elst et al., 2017). These negative emotional outcomes of remote work deplete personal resources (Ten Brummelhuis & Bakker, 2012). Similar to the findings discussed under theme 1, this finding is new to the extent that it suggests a work-related version of ‘home-sickness’ (Du et al., 2018b).

It can be argued that these findings demonstrate that that remote work is not simply about a change in work space, but that it changes the nature of work itself (Sardeshmukh et al., 2012) to the extent that it influences both personal and contextual resources (Ten Brummelhuis & Bakker, 2012), and creates conflicting work and personal life demands. However, these influences and demands tend to be paradoxical in nature and, at least to some extent, unpredictable.

### Theme 3: Working from home influences employee engagement and employee experience, but not uniformly

#### Sub-theme 3.1: Energy, enthusiasm and immersion increase for some, but decrease for others

For most participants, the attributes of working from home positively affected the dimensions of employee engagement: energy, enthusiasm and immersion (Schaufeli, 2013):

‘I sleep a bit more because I don’t have to get up so early … I think my energy is definitely a bit more, because the mind is in a good space.’ (P13, 30–40, middle management)

‘Energy levels probably increased a bit, because you are getting it [*work*] done faster.’ (P1, 30–40, middle management)

Enthusiasm was notably linked to an experience of being able to make meaningful contributions or experience the final contribution of own work:

‘I felt very valued, very important because there was a lot of focus on HR having to be involved … So, I really felt good about my job. And inspired and driven.’ (P12, 40–50, senior management)

In contrast, working from home also had the consequence of reducing the meaningfulness of jobs and even informal roles, with adverse impacts on engagement and employee experience:

‘A big part of my role has changed … I’m also the occupational health and safety [*officer*] … I’m a first aider, a floor warden and fire warden … in a way I was office mom as well. So that whole role is taken from me completely.’ (P9, 60–70, middle management)

This finding confirms the previous research finding (Lemon, 2019: Maslach & Leiter, 2005) that engagement and employee experience are associated with meaningful work, and also Kahn’s (1990) notion that psychological meaningfulness promotes engagement. Most participants reported increased levels of immersion, mostly attributed to fewer disruptions, allowing greater levels of concentration. However, immersion levels varied from day to day:

‘Definitely way more immersed in my job than I was before.’ (P1, 30–40, middle management)

‘You get immersed, but it also depends from day to day. You struggle to switch off some evenings … You are wholly fed-up, you just want to finish.’ (P3, 20–30, middle management)

This finding also confirms previous research (Masuda et al., 2017), which revealed that engagement is not a static state but fluctuates.

Paradoxically, extensive working from home also led to a decrease in employee engagement and signs of burnout, which negatively affected energy levels and increased pressure and anxiety. Decreased energy levels and increased fatigue led to a decrease in enthusiasm:

‘I have been feeling exhausted. Even now I’m feeling quite exhausted. I … felt like I just couldn’t cope anymore.’ (P8, 40–50, middle management)

‘It felt like 24/7, weekends, until two o’clock in the mornings … it drained my energy, but I just had to keep on going.’ (P7, 40–50, senior management)

This finding suggests signs of burnout, that is, a state of complete mental and emotional exhaustion (Maslach & Leiter, 2005), and confirms the finding of Sardeshmukh et al. (2012) that engagement may decrease with extensive remote working. These findings confirm how the depletion of personal resources in the face of contextual work demands (Ten Brummelhuis & Bakker, 2012) adversely influence engagement and employee experience.

#### Subtheme 3.2: Working from home influences employee experience on all three of its domains

As the themes and sub-themes are interdependent and not separable, the influence of working from home on employee experience is explicated in the preceding discussions. Without duplicating those discussions, it can be concluded that working from home significantly influenced employee experience on all three of its domains, namely, physical, cultural and technological (Morgan, 2016). This finding emphasises that remote work is not simply about providing spatial mobility but that it changes the nature of work itself, employee engagement and experiences in work processes (Sardeshmukh et al., 2012).

#### Sub-theme 3.3: Being forced to work from home influences engagement and employee experience

Participants had varied experiences regarding compulsory working from home. Especially those who were able to work from home effectively or were used to remote working did not experience significant effects and appreciated the working from home decree:

‘I was more grateful as opposed to unhappy about being forced to come and work from home.’ (P1, woman, middle management)

This finding is somewhat unique as the previous research study has emphasised that employee choice is required for remote working to show full positive outcomes (Hyatt & Coslor, 2018). However, the finding can be ascribed to the context of the COVID-19 pandemic during which people may have preferred to stay at home for safety reasons. As lockdown restrictions were decreed, these assisted participants do deal with psychological implications:

‘It helped with like guilt because there was no other option … We had no other options, so I think it helped.’ (P2, 30–40, middle management)

These findings demonstrate the role that personal factors and the social environment play in employees’ experience and the consequences of being forced to adapt to remote work.

#### Concluding reflections – Theme 3

The findings under theme 3 confirm that engagement and employee experience are associated with meaningful work (Bailey et al., 2017; Lemon, 2019), and also Kahn’s (1990) notion that psychological meaningfulness promotes engagement. It also confirms that although working from home promotes engagement in the short term, engagement may decrease with extensive remote working (Sardeshmukh et al., 2012), even to the point of burn-out (Maslach & Leiter, 2005; Wood et al., 2020). Similar to the concluding reflections under theme 2, the findings emphasise that remote work is not simply about changing spatial mobility but that it changes the nature of work to the extent that it influences both personal and contextual resources (Ten Brummelhuis & Bakker, 2012). These findings demonstrate the important role that personal factors and the social environment play in employees’ experience of working from home and the consequences of being forced to adapt to remote work.

### Theme 4: Experiences of working from home created expectations of continuing with this praxis in future

#### Sub-theme 4.1: The perceived effectiveness of virtual working created a new working norm

Enabling employees to work from home forced organisations to digitise and implement online work practices. Several participants observed how adapting to working online and virtual meetings had proved that effective remote performance was possible:

‘It has sort of now has become an acceptable norm.’ (P7, 40–50, senior management)

As virtual communication became the norm, there was a drastic improvement in all participants’ skills and online meeting capabilities, and an appreciation of the flexibility that virtual meetings allowed:

‘I can jump into a meeting like this one, I’m done in 45 minutes or an hour or whatever. And then I can carry on with work. I can work up to a minute before the meeting and a minute after the meeting.’ (P18, 30–40, executive)

The introduction to digital collaboration tools mostly had a positive impact:

‘Because the technology actually allows everybody working together on a document, it’s actually much easier now because everybody is … working on the document together. Whereas, in the office, you have to stand up and go and speak to somebody else and you don’t have everybody working together at the same time.’ (P21, 30–40, middle management)

The downside to online working was that more meetings were held and some participants spent their entire day on meetings, even after hours, resulting in ‘Zoom fatigue’ and increased irritability:

‘[*P*]eople also started getting zoomed fatigue … you were forever just on-screen in all these virtual meetings.’ (P22, 30–40, junior management)

Notwithstanding their adverse outcomes, virtual meetings and online working were experienced as surprisingly effective, creating perceptions of remote work (especially working from home) becoming the new working norm.

#### Sub-theme 4.2: Employees want to continue working from home, but not exclusively so

Despite the challenges relating to working from home, more than 70% of participants preferred working from home over office work:

‘I definitely would prefer working from home, just purely based on time wasted in traffic and the frustration and not to talk about the saving on vehicle maintenance and fuel costs.’ (P16, 40–50, junior management)

Although all participants expressed their desire to continue with working from home, only three (12%) were in favour of working from home exclusively, with the others preferring to alternate the flexibility of working from home with the structure and interaction of office work:

‘I think there should be a good balance between going to the office and working from home, not exclusively working from home.’ (P13, 30–40, middle management)

Some participants suggested the need for working from different environments as a reason for alternating between remote work and office work, as ‘living at work’ became overwhelming. All participants mentioned the need for physical interaction as a major reason to also work from the office:

‘Flexibility to be able to going in to the office, engage, but also work at home, I think is very suited and much better environment and style of working.’ (P12, 40–50, senior management)

Most participants mentioned the ability to carry out more work, the ability to concentrate and flexibility as important reasons for working from home to continue:

‘Although we still tend to work core hours, I find … bit more flexibility in general. I can pop out to the shop quickly. I can go and … do this and that and then just catch up with a few things on the weekend.’ (P5, 30–40, middle management)

Although the preferred number of work-from-home days varied between 1 and 4 days per week, more than 60% of participants preferred working from home 2-3 days a week. Other participants felt that the ideal way would be the ability to choose when to work from home or the office:

‘I would like to go to the office twice a week, and have the flexibility of choosing when I go to the office … So if my work is done, I have the option of going home or doing other stuff.’ (P19, 30–40, middle management)

Although the previous research study found that variation in working from home and office work was required to improve engagement and employee experience (Lee, 2018; Sardeshmukh et al., 2012), the findings of this study contradict the notion (Vander Elst et al., 2017) that the disadvantages of remote working exceed its advantages.

#### Concluding reflections – Theme 4

The findings under this theme suggest that working from home is largely expected to become a new working norm, at least partly so, and notwithstanding some adverse outcomes. This finding supports the outcomes of several survey polls (Iometrics & GWA, 2020; Lavelle, 2020), which suggest that working from home, at least partly, is expected to become a new norm in the way of work.

## Discussion

In this research study, working from home proved to be an effective alternative work arrangement. However, notwithstanding idealistic expectations and excitement about it, exclusive working from home could hamper employee engagement and employee experience to the point of burnout. The findings confirmed that remote working was more than just a change in physical location; it also altered the surrounding environment, resources required, organisational expectations and interactions with other employees. A multitude of factors contribute to or detract from the work-from-home experience. When job resources and organisational support exceeded job demands, this resulted in higher levels of employee engagement and positive experiences. Conversely, where job demands, such as increased workload and family commitments, exceeded resources, this resulted in adverse experiences and decreased engagement. Organisational support, a conducive organisational culture and trust were seen as key requirements of working from home, whereas a lack of face-to-face interaction was found to be a key obstacle. Other factors contributing to employee engagement and employee experience included flexibility and improved work–life balance, physical infrastructure, task meaningfulness and cost and time savings by not having to commute to and from the office. Whilst this study’s findings have confirmed contradictions regarding varying levels of disruptions at home, the study generally revealed greater productivity at home than at the office, although not for all. Working from home provided appreciated benefits at an individual level (e.g. improved work–life balance, and cost and time savings) and at an organisational level (increased productivity and employee retention).

The paradoxical findings revealed that extensive working from home inevitably came with various unwanted consequences. Whilst most participants noted their ability to manage their personal and professional lives better, they also found that blurred boundaries between work and family life made psychological detachment from working and work–life balance difficult. They experienced disadvantages, such as decreased emotional well-being and social isolation. People, being unique, were influenced differently by external factors that could either create a conducive or non-conducive work-from-home environment. The paradoxes demonstrated that remote working solutions were imperfect in isolation and required supplementary arrangements to balance the disadvantages.

The findings confirmed a preference for remote working, but not exclusively so, as face-to-face interactions remained important. Participants preferred the flexibility of being able to combine remote work with office work and the option to work remotely for 2–3 days a week, so as to obtain the best from both the options. Being forced to work from home without choice was not seen as an ideal approach, although, because of COVID-19 safety concerns, it was largely appreciated and experienced as organisational support. During lockdown restrictions, challenges, obstacles and emotional grievances were tolerated in the context of the pandemic; however, the accompanying beneficial experiences would remain in memory and drive future expectations.

## Research contribution

The COVID-19 lockdown provided a unique ‘experimental’ environment, in which organisations were placed in a laboratory-like situation where employees could experience pure, sustained work from home, without the option of going to the office or attending social gatherings. The extreme COVID-19 context provided a rare opportunity to investigate the benefits and obstacles of working from home in a real-life situation, thus eliminating idealistic fantasies or romanticism. This facilitated the extraction of the complex relationships between the paradoxical outcomes of extensive working from home – as far as could be established – for the first time. For instance, the emergence of a work-related version of ‘home-sickness’ (Du et al., 2018b) is a new finding that was specifically enabled by this unique research setting.

This research study reveals the usefulness of the JD-R framework (Schaufeli & Bakker, 2004) and W-HR model (Ten Brummelhuis & Bakker, 2012) with regard to understanding the dynamics and outcomes of working from home extensively. A key contribution of this research study is the recommendation, notwithstanding the paradoxical outcomes, that organisations continue with work-from-home arrangements, however not exclusively. Previous studies on the adoption of flexible work arrangements assumed both the willing participation and the availability of appropriate policies. Very little research exists where both the employee and the employer were forced to implement work-from-home arrangements. The study uniquely demonstrates that working from home applies to flexibility of *place*, and that remote work cannot, in practice, be dissociated from *when* employees work. The experimental study revealed that organisations should not try to force a single remote work solution but should rather seek various options that create conducive working conditions.

### Practical implications and recommendations for practice

Although exclusive working from home is not encouraged, organisations will have to consider remote policies and practices in the post-pandemic future in order to gain many consequential benefits and to address increasing remote work expectations, for instance, consider allowing employees to work remotely for 2–3 days per week, or allow employees to work from the office as and when needed. This will require a review of organisational practices, and cultural and physical support for work-from-home arrangements.

Leaders will need to develop skills to manage their employees working remotely and provide physical and mental support. If a remote work policy is adopted, expectations and deliverables should be clearly communicated and managed interactively – with clearly defined KPIs – and discussions to be held at regular intervals. Employers and managers need to be sensitive about employees’ workload as loyal, and engaged employees may overextend themselves. Work-from-home practices require managers to be aware of the pressures that cause employees to work excessive hours at home and to support employees in finding a work–life balance. It remains the manager’s responsibility to maintain the relational side of teams and to create opportunities for social interaction and collaboration. Beyond attention to operational issues, dedicated opportunities should be sought to focus on employees’ well-being.

### Limitations and recommendations for future research

Several aspects could have influenced participants’ work-from-home experience in the context of COVID-19 lockdown regulations. Some organisations might have been sceptical about the efficacy of working from home, but were forced to follow this route. This resulted in additional pressure on participants to prove that they could work from home effectively. The pandemic had much wider psychological impacts on employees than just forcing working from home, such as anxieties about personal health, job security, home-schooling and childcare. The COVID-19 pandemic forced organisations and employees on short notice to prepare for working from home and not all were ready. Initial harsh lockdown restrictions made it more challenging to manage work–life balance. Working from home and lockdown were initially regarded as temporary measures, and people were motivated to make it work to save organisations and their jobs. The fatigue of apparent long-term lockdown may have influenced the findings. The study was conducted from the perspective of employees, and the effectiveness of working from home was not considered from the organisation’s point of view. Care must thus be taken before applying the findings indiscriminately.

Similar research is recommended to explore the impact of extensive unforced remote work (whether voluntary, offered or chosen) on the engagement and employee experience of employees. Future studies should investigate remote working experiences from the perspective of employers. More research is required on how social isolation can be curbed, and how interaction and collaboration can be enhanced when a large contingent of staff works remotely. Comprehensive research is also required to study the long-term effects of blurred boundaries between work life and family life on individuals. The long-term effects of the continuance of home-schooling also require further research.

## Conclusion

Remote working trends accelerated by the COVID-19 pandemic are unlikely to disappear as employees found working from home to be both productive and attractive. Indeed, many employees may favour organisations that provide remote working opportunities. Almost all work can now effectively be carried out remotely, and employees expect work from home to continue at least partly. Organisations that want to benefit from improved employee engagement and experience should afford employees remote work opportunities, even after the end of the COVID-19 pandemic. However, the paradoxical findings of this study demonstrate that working from home represents neither a panacea nor a proverbial headache. Rather the answer to future work arrangements lies in developing a healthy balance between the physical office presence and working from home, with an appropriate organisational and managerial support.
